# Species composition of phlebotomine sand flies and bionomics of *Phlebotomus orientalis* (Diptera: Psychodidae) in an endemic focus of visceral leishmaniasis in Tahtay Adiyabo district, Northern Ethiopia

**DOI:** 10.1186/s13071-015-0849-7

**Published:** 2015-04-25

**Authors:** Araya Gebresilassie, Oscar David Kirstein, Solomon Yared, Essayas Aklilu, Aviad Moncaz, Habte Tekie, Meshesha Balkew, Alon Warburg, Asrat Hailu, Teshome Gebre-Michael

**Affiliations:** Department of Zoological Sciences, Addis Ababa University, Addis Ababa, Ethiopia; Department of Biology, College of Natural Science, Jigjiga University, Jigjiga, Ethiopia; Department of Microbiology and Molecular Genetics, The Institute of Medical Research Israel-Canada The Kuvin Center for the Study of Infectious and Tropical Diseases, Faculty of Medicine, The Hebrew University, Hadassah Medical School, Jerusalem, Israel; Aklilu Lemma Institute of Pathobiology, Addis Ababa University, Addis Ababa, Ethiopia; Department of Microbiology, Immunology and Parasitology, College of Health Science, Addis Ababa University, Addis Ababa, Ethiopia

**Keywords:** Bionomics, Population dynamics, *Phlebotomus orientalis*, Sand fly fauna, Tahtay Adiyabo, Visceral leishmaniasis

## Abstract

**Background:**

Visceral leishmaniasis (VL) is a neglected tropical disease, which is strongly associated with poverty. VL caused by *Leishmania donovani* and transmitted by *Phlebotomus orientalis* is endemic in various remote areas of north and north-west Ethiopia. The present study was designed to determine the sand fly fauna and bionomics of *P. orientalis* in the VL endemic focus of Tahtay Adiyabo district.

**Methods:**

Sand flies were collected using CDC light traps (n = 602), sticky traps (n = 9,350) and indoor pyrethrum spray catches (n = 578 house visits) from indoor, peri-domestic and agricultural field habitats between May 2011 to April 2012. All sand fly specimens collected were identified to species level and counted.

**Results:**

In total, 100,772 sand fly specimens, belonging to 25 sand fly species (nine *Phlebotomus* and sixteen *Sergentomyia*) were collected and identified. *S. africana* and *P. orientalis* made up 59.1% and 23.5% of the collected sand flies, respectively. As it could be determined from the proportion of collections from outdoor (peri-domestic and agricultural fields) and indoor locations, *P. orientalis* appears to exhibit increased exophilic behavior. The outdoor to indoor index was 79:1 on m^2^ of sticky traps. Mean density of *P. orientalis* caught was significantly higher on horizontally placed sticky traps (mean = 60 ± 14.56/m^2^/night) than vertically deployed sticky traps (12 ± 3.57/m^2^/night). The highest abundance of *P. orientalis* occurred between March and April. Through July to September, there was a sharp decline in abundance of *P. orientalis* population. Regarding climatic variables, *P. orientalis* density in light traps and on sticky traps showed a significant positive and negative association with temperature and relative humidity, respectively. However, non-significant negative correlation was observed with rainfall pattern.

**Conclusions:**

Overall, *P. orientalis* was found to be the most abundant *Phlebotomus* species, showing marked seasonal abundance that mainly peaks during the dry season (March to April). Likewise, the people in the area usually sleep in compounds during these months that potentially expose them to a high risk of peri-domestic VL transmission.

## Background

Visceral leishmaniasis (VL), caused by *Leishmania donovani*, is an important public health problem in several regions of Ethiopia, with an estimated annual incidence of 3,700 to 7,400 cases per year [1]. This systemic disease has been reported from at least 40 areas, with the most important endemic foci being the arid south‐west and the north‐west lowlands of the country bordering Kenya and Sudan, respectively [2,3]. In recent years, reports have described increasing numbers of VL cases as well as new foci of disease in the semi-arid lowlands of Tigray Regional State, northern Ethiopia [4,5]. For instance, between 2006 and 2011, 209 VL cases were treated in Tahtay Adiyabo district [5].

So far, 22 species of *Phlebotomus* have been reported in Ethiopia. Of these, the incriminated vectors of VL, from which parasites have been detected and/or isolated and identified, include *P. martini*, *P. celiae* and *P. orientalis* for *L. donovani* from the south, south-west and northern foci ([6,7]; Gebresilassie *et al.*, unpublished data). In our study area, natural infection rates of *P. orientalis* with *L. donovani* are found to be 0.51% (Gebresilassie *et al.*, unpublished data). However, detailed studies on the bionomics of sand flies in north and north-west Ethiopia in general and Tahtay Adiyabo district in particular, are very limited since the occurrence of the disease was recently recognized [4]. Knowledge on the distribution, population dynamics, and behavior of sand fly vectors contributes to understanding of where, when, and how humans become infected with *L. donovani*. Moreover, determining the abundance and seasonal dynamics of vector species are crucial for recommending sound vector management methods towards the control of VL transmission in the area. Because of the limited information available about the sand fly vector(s) involved, an extensive entomological study aimed at identifying the sand fly fauna and bionomics of *P. orientalis*, the vector of the disease, was initiated in the rural community of Tahtay Adiyabo district.

## Methods

### Study area

**Figure 1:**
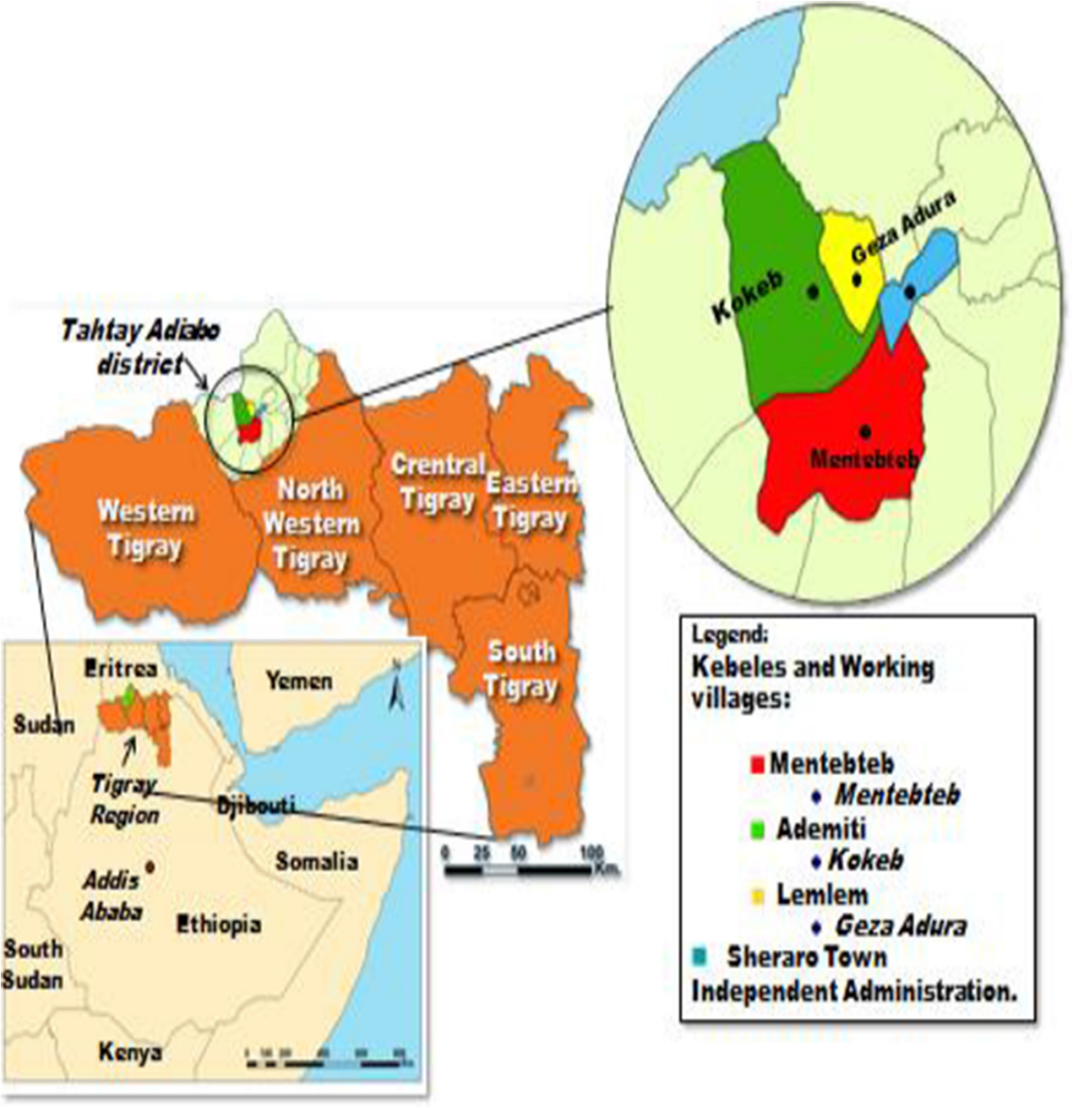
Map of Tahtay Adiyabo District (modified based on GIS of Ethiopia); red, green, and yellow colors showing selected study villages.

Longitudinal entomological investigations were undertaken in three different villages (Ademeyti, Lemlem and Mentebteb) of Tahtay Adiyabo district (14°23’41”N/37°46’15”E) in the Tigray Regional State, northern Ethiopia between May 2011 and April 2012 (Figure 1). The topography of the study area is predominantly lowland plain except in the southwest, where it is mountainous. Sheraro, the administrative center of the district, lies 1,028 meters above sea level and has a latitude and longitude of 14°23’41” N /37°46’15” E, respectively. The town is also located about 1,117 km north of Addis Ababa and 402 km north-west of Mekelle, the capital of the Regional State. The three villages were Ademeyti (14°21’31.53” N; 37°41’37.89” E; 1,060 m above sea level), Lemlem (14°22’15.27” N; 37°44’35.96” E; 1,068 m above sea level), and Mentebteb (14°19’37.78” N; 37°44’15.56” E; 1,079 m above sea level). The villages of Ademeyti and Lemlem are approximately 17 and 6 kms northwest and west of Sheraro town, respectively. The third village, Mentebteb is located about 13 km southwest of Sheraro town. The distance between the three villages is about 8–12 km.

The villages are located on hilly outcrops (peri-domestic) of sandy clay loam soil, which does not form cracks. The villages are surrounded by large fields many of which are vertisols and red clay soil. The dominant soil in the study villages is chromic vertisols [8], characterized by high contents of smectitic clay minerals. This soil type is also main agricultural field in the study area. The climate is generally characterized as tropical semi-arid area with an extended dry period of nine to ten months. The area has uni-modal rainfall pattern (July-late-September) with a mean annual precipitation of about 600 mm (Ethiopian National Meteorological Agency). During the rainy season, there is frequent cloud cover and thunder. Vertisols also become excessively muddy and even flooded during the rainy seasons. Around the last week of September the rain ceases and there begins a period marked by clear skies and cool winds which continue with gradual warming to February. March to May is the hottest part of the year with an average temperature of 39°C at noon and January is the coldest one with an average temperature of 14.2°C at night. Under the relentless sun, the vertisols dry, forming large cracks as deep as 1 m and with finer fissures below [8]. The visible moisture line recedes to the bottom of the cracks.

Three sand fly sampling habitats were considered: indoor (inside tukuls), peri-domestic habitats (homesteads shared with animal shelters), and agricultural fields on the periphery of human residence with scattered and mixed trees mainly *Acacia-Balanites-Zyzpus-Combretum* trees and some scrub vegetation. The study area was once covered with natural forest, but because of various human activities like cultivation, grazing, and wood cutting the natural vegetation have been reduced to a few scattered clumps of *Acacia-Balanites-Zyzpus-Combretum* trees/bushes. The human dwellings comprise stonewalls and wooden roofs covered with flat stones, sandy soil while few houses are made of stone wall and thatched roofs or corrugated iron roofs. The inside walls and floor of these houses are leveled with alluvial soil and plastered with mud frequently. Houses are built close to animal enclosures and are situated approximately 50–200 meters from farm fields many of which are in vertisols. The inhabitants are mainly engaged in the production of cereals and oilseeds and raising different domestic animals, cattle, sheep, and goats being predominant.

### Sand fly collection and processing

#### CDC light traps

Sand flies were collected in CDC miniature light traps (John W. Hock, Gainesville, FL) (n = 602/137 nights). Traps were set-up for three consecutive nights twice a month. These traps were used to sample sand fly species from peri-domestic and agricultural fields. No light traps were used indoors as light traps may have a tendency to attract flies from outside, and may not represent the true endophagic/endophilic species. Five CDC light traps were deployed in representative sites (i.e., on cracked walls, a stone pile produced by a collapsing hut, compound of human dwellings, and animal enclosures) of peri-domestic habitats. Likewise, another five light traps in each village were used to sample sand flies in agricultural fields, where they were suspended under trees/ bushes, in open fields, dry riverbeds, and the edge of farmlands with different bushes. CDC traps were suspended with the fan 40–50 cm above the ground level. The traps were set 1 h before sunset and collected at sunset the next morning. Then, traps containing sand flies were transported to the field laboratory, where sand flies were sorted by sex and genus and were preserved in 70% ethanol for later identification to species level.

#### Sticky trap (ST) collection

A4-sized (21 × 29 cm) white sticky traps (n = 9350/187 nights) of polypropylene sheets coated with sesame oil were used for capturing sand flies from all sampling habitats. Fifty sticky traps were divided into 10 sets each having five sheets tied together on nylon string about 50 cm apart and these were placed vertically 1 m high inside ten different houses in each village to intercept and capture any endophilic sand flies. Another 10 sets of sticky traps were randomly installed horizontally 1 m high on cracked walls (2 sets), stone piles (2 sets), and animal enclosures (6 sets) in the peri-domestic ecotopes. Another 50 STs were also deployed in agricultural fields. In this habitat, five sets of STs (5 A4 sized sheets/set) were hung vertically in a row 30 cm above the ground supported by metal pegs. Simultaneously, another five sets of STs were placed horizontally on the cracks of agriculture fields. Each morning, sand flies from STs were removed using forceps and stored in 96% ethyl alcohol in labeled vials for identification. Sticky traps were deployed for three consecutive nights twice a month.

#### Pyrethrum spray catches (PSC)

Indoor resting sand flies were sampled in the morning (6:00 to 8:00) from ten randomly selected houses by the application of pyrethrum spray catches [9] between May 2011 and April 2012 twice a month. Prior to spraying in each house, all food items and small animals were removed; the openings and eaves of windows and doors were filled with pieces of cloth, and the floor was entirely covered with white plastic sheets. After the door was closed, the pyrethrum aerosol (Roach killer, M/S Kafr EI Zayat, Egypt) was sprayed for about three to five minutes. After ten minutes of spraying, the knocked down sand flies were then collected from the white sheets using fine forceps or fine camel hair brushes and placed in tubes containing 70% ethanol for latter processing and identification.

### Mounting and identification of sand flies

Collected sand flies were mounted on microscope slides in Hoyer’s medium with their heads separate from thoraces and abdomens. Species were identified based on the morphology of the external genitalia of males and the pharynx, antennal features and spermathecae of females, using different keys, [10,11] and other publications [12].

### Age grading of wild-caught male sand flies

Age grading of wild-caught males of *P. orientalis* was conducted based on the orientation of their genitalia. The external genitalia of male sand flies undergo dextral rotation on the longitudinal body axis through 180° during the initial 24 hours of adult life to assume their mature (= rotated) position [8,13,14]. In order to make use of this easily discernable physical characteristic to identify young males, *P. orientalis* males were mounted on slides. Males with un-rotated or partially rotated external genitalia were considered active for the first night of their adult life.

#### Meteorological data

Meteorological data on maximum and minimum temperatures, average relative humidity, and rainfall of Tahtay Adiyabo district during May 2011 to April 2012 were obtained from the National Meteorology Agency of Ethiopia to assess the effect of local weather elements on the seasonal dynamics of *P. orientalis*.

### Ethical considerations

Verbal informed consent was obtained from heads of households selected for sampling sand flies from inside houses.

### Data analysis

Prior to data analysis, sand fly numbers were log-transformed [log (n + 1)] to fit normal distribution and checked for normality by Shapiro-Wilk test. When trapping data did not conform to the normal distribution, the non-parametric equivalent tests of Kruskal-Wallis and Mann–Whitney-*U* were applied. Kruskal-Wallis test was followed to compare the mean numbers of *P. orientalis* collected in the three sampling villages using CDC-LTs and STs. For non-parametric comparisons, multiple-Mann–Whitney *U*-test was used and, *P-*values were adjusted with the Bonferroni correction to adjust for the inflation of type I errors when several Mann–Whitney tests are performed [15]. For habitat preference comparisons, the equivalent non-parametric Mann–Whitney *U*-test were used. Seasonal abundance of *P. orientalis* was compared using Kruskal-Wallis-test. Pearson correlation analysis was also applied to compare the effects of mean monthly temperature, relative humidity, and rainfall on the mean number of *P. orientalis* caught per trap-night. Further, Mann–Whitney *U*-test was used for comparing the number and proportions of *P. orientalis* indoor and outdoor abundance, age-grading, sex ratio in trapping methods and comparative efficacy of different arrangements of sticky traps. Statistical analysis were considered significant when *P* < 0.05 unless stated. Statistical analyses were carried out using IBM SPSS statistics, version 20 for Windows (SPSS Inc., Chicago, IL, USA), and Microsoft® Office Excel 2007.

## Results

### Species composition and relative abundance of sand flies

In total, 100,772 sand fly specimens, belonging to twenty-five species (nine *Phlebotomus* and sixteen *Sergentomyia*) were collected and identified (Table 1). The genus *Phlebotomus* represented six subgenera while four subgenera were identified in *Sergentomyia*. Different sand fly species identified in the present study consist of *Phlebotomus* (*Larroussius*) *orientalis, P.* (*Anaphlebotomus*) *rodhaini, P.* (*Synphlebotomus*) *martini, P.* (*Phlebotomus*) *bergeroti*, *P.* (*P.*) *papatasi, P.* (*P.*) *duboscqi, P.* (*Paraphlebotomus*) *alexandri, P.* (*Parvidens*) *lesleyae*, *P.* (*Parv.*) *heischi*, *Sergentomyia (Parrotomyia) africana, S.* (*Sergentomyia*) *schwetzi, S.* (*S.*) *antennata, S.* (*S.*) *bedfordi group, S.* (*S.*) *dubia,* S. (*Sintonius*) *clydei, S.* (*Sin.*) *adleri, S.* (*Sin.*) *calcarata, S.* (*Sin.*) *subtilis, S.* (*Sin.*) *adami, S.* (*Sin.*) *satti, S.* (*Sin.*) *christophersi, S.* (*Sin.*) *capensis, S.* (*Sin.*) *thomsoni, S.* (*Sin.*) *affinis*, and *S.* (*Grassomyia*) *squamipleuris.*

**Table 1:** **Relative abundance and fauna of sand flies collected from three villages of Tahtay Adiyabo district, May 2011 to April 2012**

**Sand fly species**	**CDC light traps**	**Sticky traps**	**Indoor space sprays**	**Total**	**Relative frequency (%)**
	**M/F**	**M/F**	**M/F**		
*Phlebotomus orientalis*	5,360/2,606	14,547/1,150	26/22	23,711	23.53
*P. rodhaini*	17/13	29/33	0/0	92	0.09
*P. bergeroti*	26/20	15/13	2/3	79	0.08
*P. martini*	19/11	11/10	3/3	56	0.06
*P. papatasi*	14/16	6/4	0/3	43	0.04
*P. duboscqi*	5/9	1/6	1/5	27	0.03
*P. alexandri*	0/2	0/0	0/0	2	0.002
*P. lesleyae*	8/85	21/33	0/0	147	0.14
*P. heischi*	2/2	0/2	0/0	6	0.006
*Sergentomyia africana*	19,373/1,7812	10,301/10,423	406/1204	59,519	59.06
*S. schwetzi*	774/1,255	1,191/1,358	97/365	5,040	5.00
*S. clydei*	1,059/1,631	446/721	60/111	4,028	3.99
*S. antennata*	*346/276	*1398/1097	*74/344	3,535	3.51
*S. bedfordi*	270/388	421/402	13/32	1,526	1.51
*S. dubia*	*-/198	*-/894	*-/75	1,167	1.16
*S. squamiplueris*	146/278	129/64	6/6	629	0.62
*S. adleri*	132/168	105/122	36/48	611	0.61
*S. calcarata*	27/102	38/211	6/91	475	0.47
*S. subtilis*	4/11	11/18	0/0	44	0.04
*S. adami*	4/5	3/2	6/7	27	0.03
*S. satti*	2/0	0/0	0/0	2	0.002
*S. christophersi*	0/0	1/1	0/0	2	0.002
*S. capensis*	0/0	1/1	0/0	2	0.002
*S. thomsoni*	1/0	0/0	0/0	1	0.001
*S. affinis*	1/0	0/0	0/0	1	0.001
Total	27,590/24,888	28,675/16,565	736/2,318	100,772	100

*Males of *S. antennata* and *S. dubia* are morphologically difficult to distinguish with certainty (Abonnenc and Minter [11]).

The relative abundance of these species is indicated in Table 1. The most abundant species of *Phlebotomus* was *P. orientalis* (23.5%) followed by *P. lesleyae* (0.14%). While the other *Phlebotomus* species constituted only 0.31% of the entire sand fly collection. Among the genus *Sergentomyia*, *S. africana* (59.1%) was the most prevalent species followed by *S. schwetzi* (5%).

### Comparison of the three villages for *P. orientalis* productivity

Mean numbers of *P. orientalis* collected from the three sampling villages (Ademeyti, Lemlem, and Mentebteb) using CDC light traps and sticky traps during the entire collection period are illustrated in Table 2. Significant differences were recorded in the mean numbers of *P. orientalis* captured per CDC trap/night in the three sampling villages (Kruskal-Wallis test, *P <* 0.05). Likewise, the sampling villages significantly differed in their sand fly productivity on sticky trap collections (Kruskal-Wallis test, *P <* 0.05). In both trapping methods, Ademeyti and Lemlem were the most productive sampling villages for *P. orientalis* population compared to Mentebteb (Table 2).

**Table 2:** **Mean numbers of** ***P. orientalis*** **collected by CDC light traps and sticky traps from three different sampling villages, May 2011 to April 2012**

	**Mean number ± SE of** ***P. orientalis***	
**Sampling villages**	**CDC light traps (trap/night)**	**Sticky traps (m** ^**2**^ **/night)**
Ademeyti	6.57 ± 1.57^a^	9.84 ± 2.49a
Lemlem	4.53 ± 1.02^a^	8.58 ± 2.69^a^
Mentebteb	2.85 ± 0.58^b^	3.75 ± 1.43^b^

Mean values followed by different letters in the same column are statistically significant (Kruskal-Wallis test, *P <* 0.05).

### Habitat preference of *P. orientalis*

More mean number of male and female *P. orientalis* was caught in agricultural fields than in peri-domestic habitats in CDC light traps, although this number in both sexes in the two habitats was not significantly different (Mann Whitney *U*-test, *P >* 0.05; Table 3). Mean density of *P. orientalis* males and females captured in light traps in agricultural fields was 5.19 ± 1.86 and 1.47 ± 0.33 per trap/night (Table 3), respectively. While the mean density in peri-domestic ecotopes for *P. orientalis* male was 1.47 ± 0.33 per trap/night and 1.83 ± 0.41 per trap/night for females.

**Table 3:** **Mean numbers (± SE) of** ***P. orientalis*** **collected in CDC light traps/night from different sampling habitats, May 2011 to April 2012**

**Habitat types**	**Total number of males (mean/trap/night ± SE)**	**Total number of females (mean/trap/night ± SE)**	**Total number of (mean/trap/night ± SE)**
Peri-domestic	2.69 ± 0.81^a^	1.47 ± 0.33^a^	4.16 ± 1.08^a^
Agricultural fields	5.19 ± 1.86^a^	1.83 ± 0.41^a^	7.02 ± 2.14^a^

Mean values followed by the same letters in the same column are statistically not significant (Kruskal-Wallis test, *P >* 0.05).

Unlike light trap collections, habitat types had significant effects on the abundance of *P. orientalis* male and female populations on sticky trap captures (Kruskal-Wallis-test, *P* < 0.05; Table 4). A significantly higher mean density of *P. orientalis* males was recorded in agricultural fields (mean = 16.92 ± 3.55/m^2^ trap/night) followed by peri-domestic (mean = 3.66 ± 1.48/m^2^ trap/night) and indoors (mean = 0.05 ± 0.02/trap/night) (Table 4). Likewise, more mean number of *P. orientalis* females were collected in agricultural fields and peri-domestic habitats (Table 4). However, fewer mean numbers of females were found indoor (mean = 0.04 ± 0.01/ m^2^ trap/night).

**Table 4:** **Mean density (±SE) of** ***P. orientalis*** **collected per m** ^**2**^ **sticky trap/night from different sampling habitats over one, May 2011 to April 2012**

**Habitat types**	**Total no. males (mean/m** ^**2**^ **/night ± SE)**	**Total no. females (mean/ m** ^**2**^ **/night ± SE)**	**Total no. (mean/ m** ^**2**^ **/night ± SE)**
Indoor	0.05 ± 0.02^a^	0.04 ± 0.01^a^	0.08 ± 0.03^a^
Peri-domestic	3.66 ± 1.48^b^	0.7628 ± 0.20^b^	4.43 ± 1.60^b^
Agricultural fields	16.92 ± 3.55^c^	1.2972 ± 0.23^c^	18.22 ± 3.70^c^

Mean values followed by different letters in the same column are statistically significant (Kruskal-Wallis test, *P <* 0.05)

### Indoor resting behavior of *P. orientalis*

Table 5 shows the numbers of *P. orientalis* and other sand flies found resting at 6:00 to 8:00 hours in rooms. Of 3,054 indoor resting sand flies captured in the pyrethrum spray collections during 578 house visits, 48 (1.57%) *P. orientalis* were found resting inside houses. In particular, female populations of *P. orientalis* had low endophilic behavior, constituting only 0.95% of the entire indoor resting specimens collected. Among *Sergentomyia* spp. *S. africana* had a greater proportion (52.72%) followed by *S. schwetzi* (Table 5).

**Table 5:** **Indoor resting sand fly species captured inside human dwellings using pyrethrum spray collections, May 2011 to April 2012**

	**Sand fly specimens collected per 578 house visits**		
**Sand fly species**	**Male (%)**	**Female (%)**	**Total (%)**
*P. orientalis*	26 (3.53)	22 (0.95)	48 (1.57)
*P. duboscqi*	1 (0.13)	5 (0.22)	6 (0.20)
*P. bergeroti*	2 (0.27)	3 (0.13)	5(0.16)
*P. martini*	3 (0.40)	2 (0.09)	5(0.16)
*P. papatasi*	0 (0)	3 (0.13)	3 (0.09)
*S. africana*	406 (55.16)	1,204 (51.94)	1,610 (52.72)
*S. schwetzi*	97 (13.18)	365 (15.75)	462 (15.13)
*S. antennata*	74 (10.05)	344 (14.84)	418 (13.69)
*S. clydei*	60 (8.15)	111 (4.79)	171 (5.60)
*S. calcarata*	6 (0.82)	91 (3.92)	97 (3.18)
*S. adleri*	36 (4.89)	48 (2.07)	84 (2.75)
*S. dubia*	0 (0)	75 (3.24)	75 (2.46)
*S. bedfordi*	13 (1.77)	32 (1.38)	45 (1.47)
*S. adami*	6 (0.82)	7 (0.30)	13 (0.43)
*S. squamiplueris*	6 (0.82)	6 (0.26)	12 (0.39)
Total	736	2,318	3,054

### Indoor and outdoor abundance of *P. orientalis*

In total, 52 (29 males and 23 females) from indoors and 15,901 (14,519 male; 1382 female) from outdoors were captured on sticky traps. The result of the outdoor (peri-domestic and agricultural fields combined) *P. orientalis* captures was much greater than, the indoor (Mann Whitney *U*-test, *P <* 0.05). *P. orientalis* was abundant outdoors and had low abundance indoors with a ratio of 79:1 on m^2^ of sticky traps (Table 6).

**Table 6:** **Indoor and outdoor abundance of** ***P. orientalis*** **determined by sticky traps in Tahtay Adiyabo district, May 2011 to April 2012**

**Habitat type**	**Total no. males (mean/m** ^**2**^ **trap/night ± SE)**	**Total no. females (mean/m** ^**2**^ **trap/night ± SE)**	**Total no. (mean/m** ^**2**^ **trap/night ± SE)**
Indoor	29 (0.05 ± 0.02)^a^	23 (0.04 ± 0.01)^a^	52 (0.08 ± 0.03)^a^
Outdoor*	14,519 (5.72 ± 1.31)^b^	1,382 (0.58 ± 0.10)^b^	15,901 (6.30 ± 1.39)^b^

Mean values followed by different letters in the same column are significantly different (Mann Whitney *U*-test, *P* < 0.05).

*Outdoor (combined collections of peri-domestic and agricultural field).

### Population dynamics of *P. orientalis*

**Figure 2:**
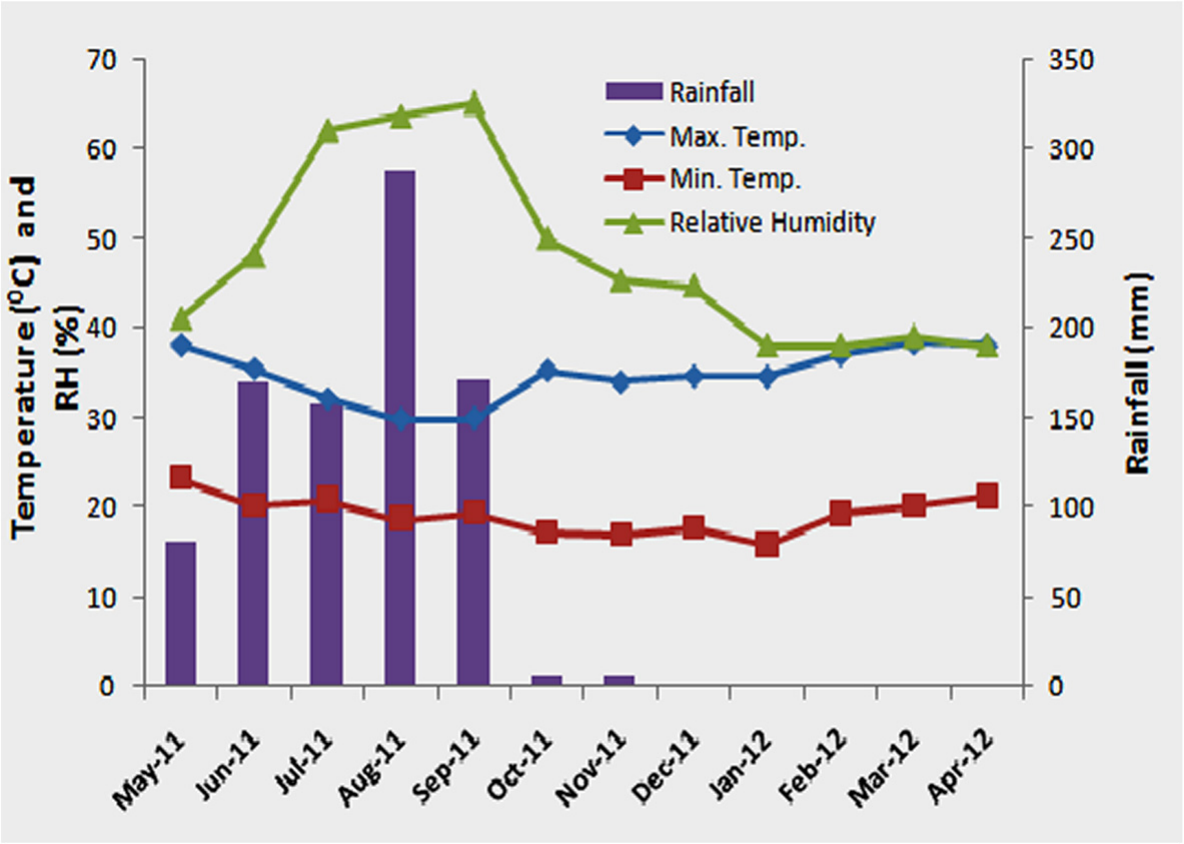
Seasonal fluctuations in the mean monthly maximum and minimum temperatures, relative humidity, and rainfall in the study area, May 2011 to April 2012.

The annual mean maximum and minimum temperatures were 38.4°C in March and 15.8°C in January, while relative humidity was 63.5% in August and between 38-39% during the months of January to April 2012, respectively (Figure 2). The main rainy period was between June and September. The maximum precipitation recorded in the area during sand fly sampling was 287.5 mm in August 2011 (Figure 2).

**Figure 3:**
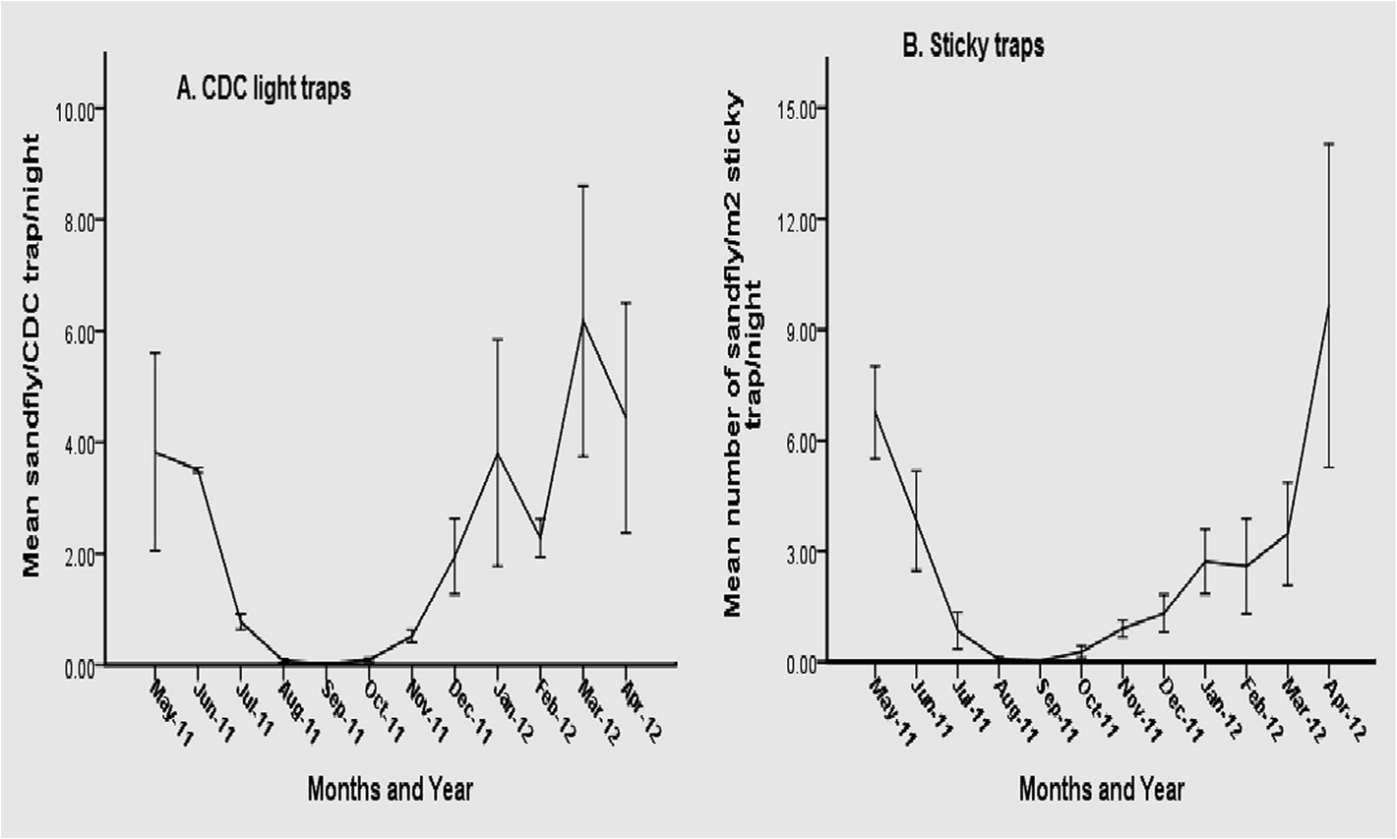
Seasonal density of *P. orientalis* collected from three villages of Tahtay Adiyabo district, May 2011 to April 2012. **A**: collected by CDC light traps (specimen/trap/night). **B**: collected by sticky traps (specimens/m^2^ trap/night).

To assess the overall seasonal changes in abundance of the *P. orientalis* in the district collected by means of CDC-light traps and sticky traps, data from each village were pooled. There were significant mean monthly fluctuations in the numbers of *P. orientalis* caught using light traps and sticky traps from different biotopes over the twelve months of trapping period (Kruskal-Wallis test, *P* < 0.05). *P. orientalis* showed distinct seasonality, with the greatest overall abundance between January and June, reaching its peak density (6.18 ± 2.43/trap-night) in March when the average temperature was also high (30.4°C). From July to December, including the rainy season (July-September) and shortly after that (October-December), there was a sharp decrease in abundance of *P. orientalis* (Figure 3A) with an increase in relative humidity and decrease in average temperature (Figure 2). The lowest mean density of *P. orientalis*/trap/night was found to be in the period from August to October in the range of 0.07 ± 0.04 to 0.09 ± 0.03.

The highest mean monthly density of *P. orientalis* recorded on sticky traps was in April (9.65 ± 4.37/m^2^ trap/night) while the lowest was during August to October, which ranged from 0.07 to 0.27/m^2^ trap/night (Figure 3B).

In light trap catches, monthly abundance of *P. orientalis* had a significant positive correlation with temperature (*r* = 0.762; *P* = 0.004). In contrast, the mean monthly abundance of *P. orientalis* was correlated negatively with relative humidity (*r* = −0.803; *P* = 0.037) and rainfall (*r* = −0.467), respectively though the relationship with the later was not significantly different (*P* = 0.126). Mean monthly density of *P. orientalis* on sticky traps had a positive correlation with temperature (*r* = 0.867; *P = 0.000*), and was associated negatively with relative humidity (*r* = −0.780; *P* = 0.003) and rainfall (*r* = −0.467; *P* = 0.163).

### Sex ratios

Sex ratios (males: females) for different sand fly species demonstrated that males caught by all methods was higher than that of females (57,001 male: 43,771 female), with an overall sex ratio of 1.3:1. For *P. orientalis*, the sex ratio in light traps was 2.1:1, which did not show any significant difference between sexes (Mann Whitney *U*-test, *P >* 0.05) as opposed to a very high ratio of male to female (12.7:1) on the sticky traps that was clearly significant (*P* = 0.000).

### Age grading of wild-caught male sand flies

Sticky traps intercepted 655 *P. orientalis* males with un-rotated or partially rotated genitalia during the study period (Table 7). Of these, 187 and 468 were captured in peri-domestic and agricultural field habitats, respectively. The difference in the proportion of *P. orientalis* immature males with un-rotated or partially rotated genitalia captured in agricultural field versus peri-domestic habitat was significant (Mann Whitney *U*-test, *P* = 0.015). However, no freshly emerged *P. orientalis* males were recorded with other collection methods used.

**Table 7:** ***Phlebotomus orientalis*** **young males (=un-rotated genitalia) caught over 12 months on sticky traps that were placed in peri-domestic and agricultural field**

**Habitat type**	**No. collected (%)**	**Mean no. ± SE/sticky trap/month**
Peri-domestic	187 (28.55)	15.58 ± 6.75^a^
Agricultural field	468 (71.45)	39.00 ± 4.98^b^
Total	655	

Mean values followed by different letters in the same column are significantly different (Mann Whitney *U*-test, *P* < 0.05).

### Comparative efficacy of sticky traps deployed horizontally versus vertically

**Figure 4:**
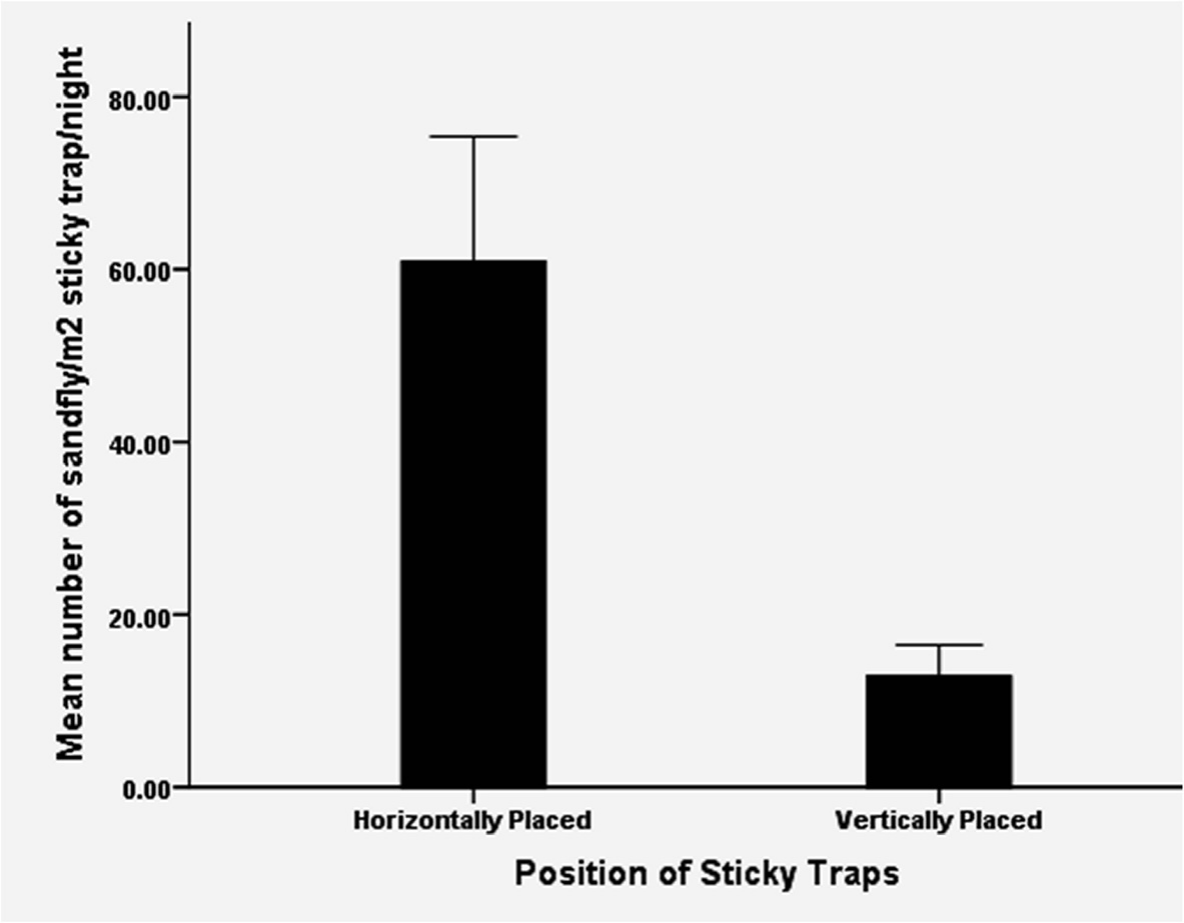
Comparison of efficacy of sticky traps deployed in different positions for trapping *P. orientalis* in agriculture field, May 2011 to April 2012.

Significant difference was observed in the mean density of *P. orientalis* captured between horizontally (HSTs) and vertically (VSTs) placed sticky traps (Mann Whitney *U*-test, *P <* 0.05). A relatively higher mean density of *P. orientalis* was found on horizontally placed sticky traps (mean = 60 ± 14.56/m^2^/night) than vertically deployed (mean = 12 ± 3.57/m^2^/night, Figure 4).

## Discussion

In the remote rural villages of Tahtay Adiyabo district, entomological studies revealed the presence of twenty five species of phlebotomine sand flies, including nine species of *Phlebotomus* (six subgenera) and sixteen species of *Sergentomyia* (four subgenera). The sand fly fauna in the area is composed of the Afrotropical elements [10,16,17].

Out of the nine species of *Phlebotomus* caught, *P. orientalis* was the most prevalent, accounting for more than 98%. This species is the proven vector of *L. donovani*, the causative parasite of VL, in Sudan, South Sudan, south western Ethiopia [6,18] and a vector in northern Ethiopia ([19]; Gebresilassie *et al.*, unpublished data). The preponderance of this species in sand fly catches was also noted in previous studies in north-west Ethiopia [19,20] as well as in the same district [21-23]. *P. martini* and *P. rodhaini* were also recorded in the present study: the former being the major vector of VL in southern Ethiopia [7] and the later was implied as possible vector *of L. donovani* between animal reservoir hosts in Sudan [24]. The three sympatric species (*P. papatasi, P. bergeroti* and *P. duboscqi*) [19] were also found, where their epidemiological role as possible vectors of zoonotic cutaneous leishmaniasis would be minor despite *L. major* from blood samples was reported in this area [4]. Among the *Sergentomyia* spp., *S. africana* was found to be the predominant species (77.5%) followed by *S. schwetzi* (6.6%). In general, the species composition of phlebotomine sand flies encountered in the present study concord with previous reports in other parts of Ethiopia [22,23,25,26].

Habitat preferences of most sand fly species is associated with biotopes, harboring high levels of stable mild temperatures and high humidity, and that contain decaying organic matter allowing better breeding sites and more suitable diurnal resting shelters [27,28]. In this study, more number of *P. orientalis* was collected in agricultural fields than other habitats. Agricultural fields are mostly vertisols, which are characterized by high contents of clay minerals that enhance swelling when hydrated and shrinkage upon desiccation, thereby, causing extensive cracking during the dry season [8]. The combination of high humidity and stable temperatures maintained throughout the dry season, and the availability of organic matter that provide food for larval development in the deeper layers of cracked vertisols [8,27], possibly explains the relatively large populations of *P. orientalis* in cultivated fields than other habitats observed. Secondly, the presence of trees and scrub vegetations provide shade and source of sugar for adult populations [29]. Earlier studies in various parts of Sudan also stressed that *P. orientalis* is mainly associated to forest area with large expanses of vertisols [10,30,31] and rarely associated with human dwellings.

Exophilic and endophilic behaviors of sand flies are important from the control point of view and determining where transmission takes place. It is evident from the study that *P. orientalis* exhibited strong exophilic behavior expressed in outdoor/indoor index of 79:1 on m^2^ of sticky traps. Supportive of the sticky trap indoor collections, less proportion of *P. orientalis* females were found resting indoors in 578 house visits, indicating lower rates of endophily. Elnaiem *et al.* [31] reported the same behavior in Umsalala village in eastern Sudan. Indoor collections of *P. orientalis* in some villages of north-west Ethiopia also yielded less capture (Aklilu, unpublished data). As the weather, in Tahtay Adiyabo is typically warm from January through May, the people usually sleep outdoors in their compounds during these months. Farmers also usually keep domestic animals in the yard overnight often within a few meters of the sleeping area, a practice that contributes to an increased abundance of *P. orientalis* females. Therefore, these activities expose people to the bite of *P. orientalis* leading to an outdoor transmission of *L. donovani*.

Our data demonstrated that *P. orientalis* in Tahtay Adiyabo appear to increase in density during the hot-dry period of March to April. A similar trend was also reported in Sudan, where the numbers of *P. orientalis* captured using sticky traps and CDC light traps remained low in the early dry season (January and February) and increased highly between March and May, after the hot, dry weather has begun [10,20,32,33]. Likewise, it was found that *P. orientalis* appeared to reach its peak during the driest months of March and April in Kafta-Humera, north-west Ethiopia [20,34]. Importantly, this sand fly period also tends to be the most likely period of VL transmission in humans as females was found with 0.51% rate of natural infection with *L. donovani* in the area (Gebresilassie *et al.*, unpublished data). The current study also demonstrated that the adult populations of *P. orientalis* diminish and disappear as the rain commences. This population disappearance was also observed in preceding studies in southern Sudan [10]. However, our findings did not concur with the previous studies in north-west Ethiopia [34] and in eastern Sudan [31].

Sand fly population dynamics is largely regulated by a complex interplay between the biotic potential of the vector species and meteorological-environmental conditions [35-37]. In the current study, temperature and relative humidity showed significant correlations with *P. orientalis* abundance. Sharp increases in population density of *P. orientalis* from March to April coincide with an increase in temperature and a reduction in relative humidity in the area. Presumably, the rapid decrease in abundance of *P. orientalis* through July to October largely attributed to an increased amount of rainfall that completely floods the surface and seals the deep cracks of vertisols, leading to a micro-climate change in the breeding/resting sites of the fly population. Moreover, changes in the monthly temperature and relative humidity during those periods might have resulted in a decrease in population abundance. During the rainy season, *P. orientalis* seems to undergo diapauses as larvae, which were observed in the fourth larval stage of laboratory colony [38]. The diapause is possibly broken by the end of rainy season as temperature increases re-opening of soil cracks.

As for the sex ratio of sand flies throughout the collections, *P. orientalis* had higher proportion of male to female both in CDC light traps and sticky traps, though the mean number ratio of male to female for the former was not significant. This male biased sex ratio was also observed for other sand fly species [36,39,40]. For instance, Kasap *et al.* [40] reported that male sand flies composed of 80% of their collections conducted in an endemic CL. Given a normal sex ratio of 1, then skewed ratios in favour of males might be related to the fact that the traps were placed near emergence sites, where males are generally abundant.

The natural breeding habitats of sand flies could be determined from sticky trap catches as these traps usually capture sand flies by passive interception rather than attraction in their diurnal/breeding habitats [41]. In the present study, sticky traps deployed in vertisols captured higher percentages of freshly emerged males of *P. orientalis*. This habitat constituted more than 70% of *P. orientalis* immature males caught with either un-rotated or partially rotated genitalia. More accurate recording of the condition of males with un-rotated external genitalia can indicate the proximity of immature sites [42]. Moreover, males apparently are more sedentary than females [43,44]. Deeply cracked vertisols in open cultivated fields and tree-related habitats were also identified as breeding sites for *P. orientalis* in our study area [8]. Accordingly, it can be conceived that a relatively high catch with large proportion of males with un-rotated or partially rotated genitalia in our study indicates a proximity to breeding sites.

Significantly, higher mean density of *P. orientalis* was recorded on horizontally placed (HSTs) STs than vertically (VSTs) deployed. Both sexes of *P. orientalis* were attracted to HSTs, albeit twenty two times as many males as females were trapped. Interestingly, deploying STs horizontally can be a practical solution for monitoring sand fly populations, particularly in flat plains with crevice although it remains to be determined in other sand fly habitats. The higher proportion of male *P. orientalis* on HSTs in the present study could be due to the attraction behavior of this vector species to the horizontal surface, establishing mating swarms [21,45]. Alternative reason could be the difference in the flight angle of the species.

## Conclusions

In conclusion, our study demonstrated that *P. orientalis* was found to be the most abundant *Phlebotomus* species, showing distinct seasonality that mainly peaks during the dry season (March to April) within the entire study area. Furthermore, this study depicts the exophilic behavior of *P. orientalis*. This behavior is of practical importance because it apparently makes the species less vulnerable to insecticide residual spraying (IRS) or long lasting insecticidal nets in indoor conditions. However, the small number of *P. orientalis* collected in sticky traps and pyrethrum spray catch indoors may still be of epidemiological significance. Therefore, control programs designed to contain VL transmission in different villages of Tahtay Adiyabo should focus mainly on targeting *P. orientalis* in outdoors without ignoring its minor endophilic behavior.
